# Acetabular rim extension using a personalized titanium implant for treatment of hip dysplasia in dogs: short-term results

**DOI:** 10.3389/fvets.2023.1160177

**Published:** 2023-04-20

**Authors:** Irin Kwananocha, Joëll Magré, Koen Willemsen, Harrie Weinans, Ralph J. B. Sakkers, Thijs How, Femke Verseijden, Marianna A. Tryfonidou, Bart C. H. van der Wal, Björn P. Meij

**Affiliations:** ^1^Department of Clinical Sciences, Faculty of Veterinary Medicine, Utrecht University, Utrecht, Netherlands; ^2^Research and Academic Service, Faculty of Veterinary Medicine, Kasetsart University, Bangkok, Thailand; ^3^Department of Orthopedics, University Medical Center Utrecht, Utrecht, Netherlands; ^4^3D Lab, Division of Surgical Specialties, University Medical Center Utrecht, Utrecht, Netherlands; ^5^Diergeneeskundig Specialisten Centrum Den Haag, The Hague, Netherlands

**Keywords:** hip dysplasia, shelf arthroplasty, 3D printed implant, femoral head coverage, acetabular rim extension, dog

## Abstract

Hip dysplasia (HD) is a common orthopedic problem in young dogs. To decrease the laxity of the hip joint related to HD, the surgical treatments are recommended to increase femoral head coverage. ACEtabular rim eXtension (ACE-X) using a personalized 3-dimensional printed titanium shelf implant is a new surgical treatment to increase femoral head coverage and decrease laxity of the dysplastic hip joint, however, the efficacy is less know. Client-owned dogs older than 6 months with clinical signs of coxofemoral joint subluxation and radiographic evidence of HD with no or mild osteoarthritis (OA) were included. The Norberg angle (NA), linear percentage of femoral head overlap (LFO), and percentage of femoral head coverage (PC) were investigated radiographically and with computed tomography (CT) before and after surgery. OA was graded (scores 0–3) according to the maximum osteophyte size measured on CT. In addition, joint laxity (Ortolani) test results, gait analysis, and the Helsinki chronic pain index (HCPI) questionnaire were obtained at preoperative, immediately postoperative and at 1.5- and 3-month evaluations. Acetabular rim extension was performed in 61 hips of 34 dogs; NA, LFO, and PC were significantly higher immediately postoperatively and at the 1.5- and 3-month follow-up examinations compared with preoperative values (*p* < 0.05). Osteophyte size gradually increased over time (*p* < 0.05). The OA score significantly increased between preoperatively and directly postoperatively, and between preoperatively and at 3-month follow-up (*p* < 0.05). The laxity test normalized in 59 out of 61 hips after surgery, and the HCPI questionnaire showed that the pain score decreased significantly at 1.5 and 3 months, postoperatively. The force plate showed no significant improvement during the 3 months follow-up. Although pain reduction by the implant was unclear in short-term results, a personalized shelf implant significantly increased femoral head coverage and eliminated subluxation of the dysplastic hip joint. Further studies are required to study the long-term efficacy of gait, chronic pain, and progression of osteoarthritis.

## Introduction

Hip dysplasia (HD) is one of the most common orthopedic problems in young dogs with a prevalence of 0–73.4% depending on the breed (1). HD is a consequence of the disparity between primary muscle mass and disproportionately rapid skeletal growth, which leads to hip joint laxity (2–4). Laxity of the hip joint can lead to hip pain, progressive osteoarthritis (OA), and limb dysfunction (5). To decrease the development of hip OA, early treatment of HD is recommended.

Although non-surgical management consisting of lifestyle changes including body weight management, medication, physiotherapy, and exercise modification, will improve clinical signs related to HD in most dogs in the short term, the improvements are rarely maintained in the long term (6, 7). Currently, several joint-preserving surgical techniques are used to increase the coverage of the femoral head in young dogs with HD. These techniques include juvenile pubic symphysiodesis (JPS), double or triple pelvic osteotomy (DPO/TPO), and shelf arthroplasty or dorsal acetabular rim (DAR) arthroplasty (DARthroplasty) (8–12). JPS is mostly a prophylactic procedure in dogs younger than 5 months old. It involves using electrocoagulation to induce premature closure of the pubic symphysis. This procedure results in ventrolateral rotation of the acetabular rim which increases the acetabular angle by 12–21 degrees at 1 year of age when the surgery is performed at 12–24 weeks old (9, 13).

Double and triple pelvic osteotomy (DPO/TPO) are both procedures that are designed to increase acetabular ventroversion to increase femoral head coverage and minimize femoral head subluxation in dogs with excess hip laxity. TPO involves periacetabular osteotomies of the ilium, pubis, and ischium. After completion of the osteotomies, an ilial bone plate that corresponds to the desired amount of rotation is selected and applied. Despite modifications to the technique and the use of new plates, the postoperative complication rate of TPO ranges from 7 to 70% (14–17). In an effort to simplify TPO and reduce complications after TPO, a modified technique called DPO was developed. DPO is a variation that differs from the more traditional TPO in that the ischium is not cut resulting in a less mobile caudal iliac segment and preservation of pelvic geometry. Although the postoperative complication rates of DPO decreased to 8–21% (8, 10), this technique is invasive with extensive learning curves. Additionally, in bilateral hip dysplastic dogs, DPO/TPO is often performed in two separate sessions with a few months in between, thereby prolonging rehabilitation time and facilitating the progression of osteoarthritis in the untreated hip (10, 11, 18). Shelf arthroplasty or DARthroplasty is the procedure wherein either autologous corticocancellous bone grafts obtained from the iliac wing or synthetic materials such as a biocompatible osteoconductive polymer were placed on top of the hip capsule to extend the acetabular rim. By extending the acetabular rim, dorsal subluxation and lateral translation of the hip joint were reduced (3, 4, 19–22). These shelf arthroplasty procedures, although clinically effective, were considered suboptimal, as they do not provide consistent full coverage of the femoral head due to uncontrolled bone formation and/or have no long-term viability due to lack of osteointegration (3, 4, 23).

As a novel application of shelf arthroplasty, Willemsen et al. (24, 25) reported a patient-specific 3-dimensional (3D) printed titanium shelf implant for ACEtabular rim eXtension (ACE-X). The implant was designed according to computed tomography (CT) imaging of the pelvis and hip joints of individual dogs to optimally extend the dorsal acetabular rim and provide adequate coverage of the femoral head. In a proof-of-concept pilot study, the ACE-X implant diminished hip laxity and restored coverage of dysplastic hip joints in three clinically unaffected dogs without complication (24, 25). However, the short-term efficacy and safety of this novel patient-specific titanium shelf implant in clinically affected dogs suffering from HD remained to be investigated.

In the present study, ACE-X was performed in a cohort of young and adult with clinical signs of hip laxity due to HD. Intake and outcome results were assessed 3 months after surgery. It was hypothesized that ACE-X will increase femoral head coverage, diminish hip laxity, and relieve the pain of the dysplastic hip joints in clinically affected dogs in short-term monitoring.

## Materials and methods

### Study design

The study was designed as a prospective descriptive study, non-randomized, unblinded, self-controlled clinical trial with client-owned dogs and approved by the Veterinary Clinical Studies Committees (VCSC), Utrecht University, Utrecht, the Netherlands. The dogs remained under the care of their owners during the study period. The owners were informed about the purpose of the study, surgical ACE-X procedure, alternative treatment (e.g., conservative management or TPO/DPO treatments), treatment plan, and all potential complications (e.g., infection, implant failure, neurological deficits, and others) related to ACE-X treatment before they signed the consent form. Informed written consent was obtained from all owners prior to their dogs being entered into the study.

### Study overview

Before implantation of the implant and during the 3-month follow-up period, clinical observation, imaging, force plate gait analysis, and the Helsinki chronic pain index (HCPI) questionnaires were conducted (Table 1).

**Table 1:** Study outline in months (m).

**Evaluations**	**Preoperatively (pre-op)**	**Postoperatively (*T* = 0)**	**1.5-month follow-up (*T* = 1.5 m)**	**3-month follow-up (*T* = 3 m)**
Radiograph	x^a^	x	x	–
CT scan	x^a^	x	–	x
Osteoarthritis scoring	x	x	-	x
Force plate gait analysis	x	–	x	x
Helsinki chronic pain index questionnaire	x	–	x	x
General health assessment, orthopedic examinations, joint laxity testing	x	x	x	x

^a^The date of the preoperative radiograph (inclusion) was usually earlier than the date of preoperative CT (planning for 3D printing of the shelf implant).

### Animals

Client-owned dogs older than 6 months with clinical signs related to hip dysplasia such as lameness, exercise intolerance, swaying gait, and positive joint laxity (Ortolani) testing with no or minimal evidence of radiographic OA (OA score 0–1) (26) were included in this study between December 2019 and March 2022. Dogs of either sex and any breed were eligible to be enrolled in this study. For inclusion and exclusion, the OA from preoperative radiographs was considered and graded according to the osteophyte size as described below in the Osteoarthritis Progression section. The exclusion criteria for study enrolment were an open acetabular growth plate [in puppies < 6 months old (1)], negative joint laxity testing, moderate to severe OA (OA score 2–3) (26), systemic disease, neurological deficits, and prior hip surgery. The minimum period between previous orthopedic surgery other than hip and ACE-X was 6 weeks. Before the start of the study, dogs had a wash-out period of analgesics for at least 4 days.

### Radiographic and clinical assessment

**Figure 1:**
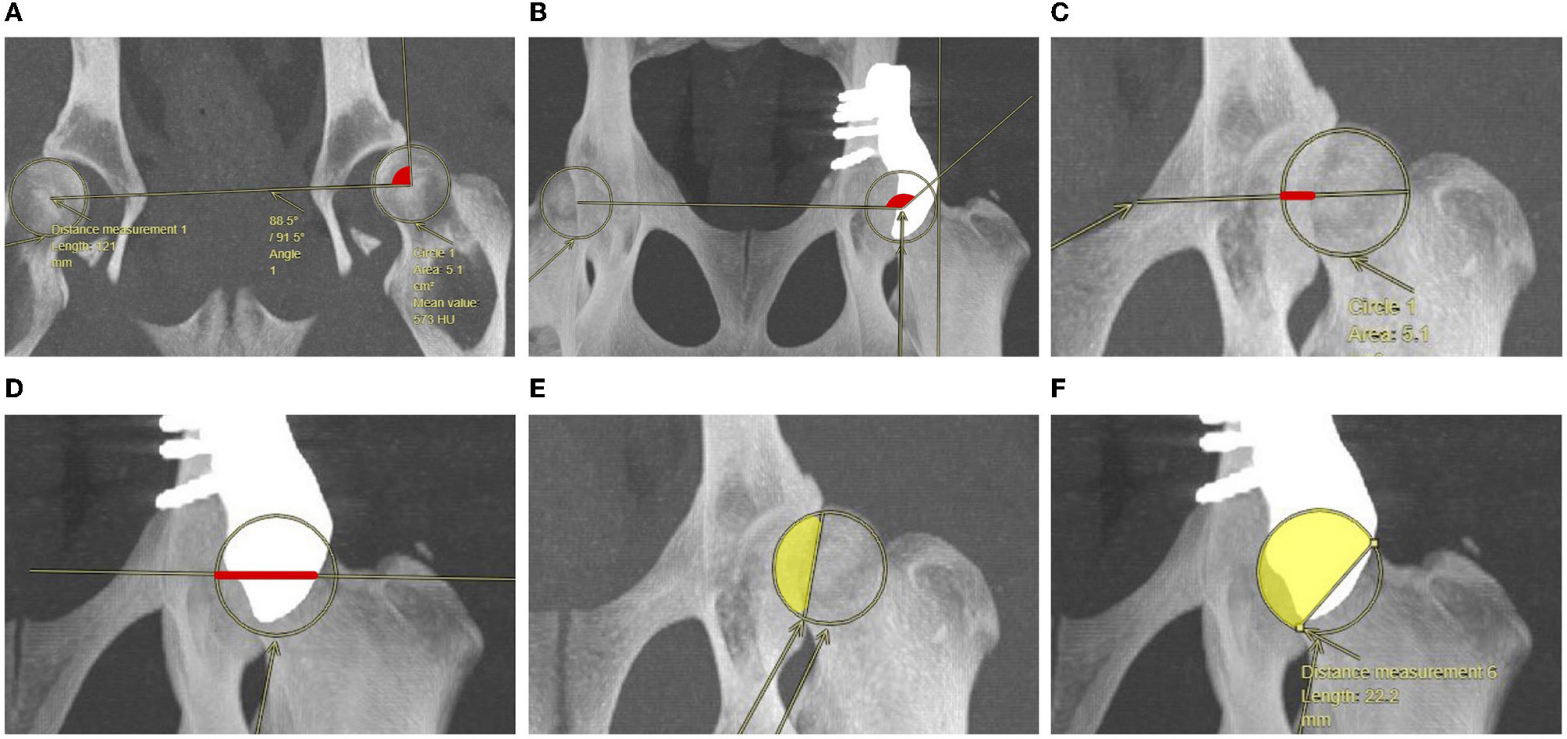
Representation of Norberg angle **(A, B)**, percentage of linear femoral head overlap (red line) **(C, D)** and percentage of femoral head coverage (yellow area) **(E, F)**, as measured in coronal CT images compared between before **(A, C, E)** and after implantation of a titanium shelf implant for acetabular rim extension **(B, D, F)**.

Clinical exam included full orthopedic examination and hip joint laxity testing using the Ortolani test under sedation. Imaging was performed using both radiography and CT. Ventrodorsal hip-extended digitized radiographs of all dogs either present at referral or taken at baseline were categorized into five groups (A to E) by national HD screening panelists (BM and MT) based on the Fédération Cynologique Internationale (FCI) scoring protocol for canine hip dysplasia (27–30). CT scans (64-slice, Siemens SOMATOM Definition AS, Siemens Medical Solutions USA, Inc.) of both hip joints in extension were performed as described previously by Lopez et al. (31). CT images were obtained with the following parameters: 120 kV, 250 mAs, 1,000 ms tube rotation time, 0.6 mm slice thickness, 0.35 spiral pitch factor, and 512^*^512-pixel matrix. The radiographs and coronal CT images of both hip joints were used to measure Norberg angle (NA), linear percentage of femoral head overlap (LFO), and percentage of femoral head coverage (PC) as well as OA score (Figure 1). All radiographic measurements were evaluated by a single observer (IK) using either Xero Viewer software for DICOM files or IC Measure software for JPEG or PDF files.

#### Norberg angle

The NA was measured as the angle between a line connecting both femoral head centers (FHCs) and a line from the FHC to the most (cranio) lateral point of the acetabular rim or implant rim that was outside of the femoral head circle (32, 33). If there was no single point, the most cranial part of the lateral line was chosen. For NA measurement, the CT images were adjusted to 10–50 mm slice thickness (Figures 1A, B).

#### Linear percentage of femoral head overlap

The LFO was measured by drawing a diameter line through the center of the femoral head and perpendicular to the craniocaudal axis. The LFO is measured as the percentage of the diameter line that shows femoral head coverage by the acetabulum or implant (red line) (33). For LFO measurement, the CT images were adjusted to 20–50 mm slice thickness (Figures 1C, D).

#### Percentage of femoral head coverage

The PC was calculated as the percentage of the total femoral head area which was covered by the acetabulum or implant (31). To determine this percentage, the femoral head circle is drawn. The area within this circle is considered 100%. Then, a line was drawn connecting the most lateral edges of the dorsal acetabular rim or implant rim. The area in the circle and medial from this line is the percentage coverage (yellow area). The area is calculated with the following formula, where *S* is the area of the smallest circle segment; *A* is the area of the femoral head circle, and *l* is the length of the line that crosses the circle. For PC measurement, the CT images were adjusted to 5–50 mm slice thickness (Figures 1E, F).


$$\begin{array}{l} S=\;\frac{1}{2}\sqrt{\frac{A}{\pi }}*\left(2\sin^{-1}\left(\frac{l}{2\sqrt{\frac{A}{\pi }}}\right)-sin\left(2\sin^{-1}\left(\frac{l}{2\sqrt{\frac{A}{\pi }}}\right)\right)\right) \end{array}$$

#### Osteoarthritis progression

Osteoarthritis severity was scored by measuring the size of osteophytes on 2D CT images. OA was graded into 0 (no osteophytes present), 1 (osteophyte size < 2 mm), 2 (osteophyte size 2–5 mm), or 3 (osteophyte size > 5 mm) according to the maximum thickness (26). To avoid metal scattering of the implant at the cranial acetabular rim on CT, the osteophyte size was measured from three different positions (e.g., femoral neck and cranial and caudal acetabular rim) and from different sections of preoperative and postoperative CT in both coronal and transverse planes to find the maximum size of osteophyte before scoring OA.

### Implant design

**Figure 2:**
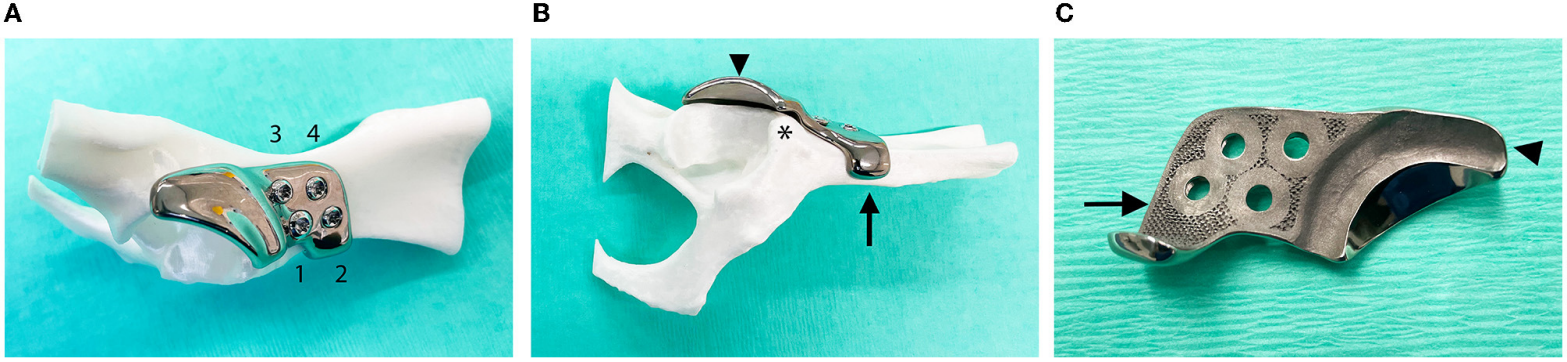
Photographs of the titanium shelf implant for acetabular rim extension. **(A)** The implant was fixed on the right ilium of a 3D-printed plastic model of a dog's pelvis using one cortical screw (1) and three locking screws (2–4). **(B)** Ventral ilium flange (arrow) of the attachment part cranial to the insertion of the rectus femoris muscle (*) improved positioning and stabilization of the implant. The acetabular rim extension part (arrowhead) of the implant increased femoral head coverage. **(C)** The bone-facing back side of the implant showed the porous surface (arrow) at the attachment part and offset (arrowhead) of the acetabular rim extension part.

The titanium implants were designed at University Medical Centre Utrecht from preoperative CT images of the complete pelvis as described by Willemsen et al. (24), using Mimics (version 24, Materialise, Leuven, Belgium) for segmentation of the DICOM files and 3-Matic (version 16, Materialise, Leuven, Belgium) for implant design. Like the implants described by Willemsen et al. (24, 25), the implants consisted of two subsections as follows: the implant–bone interface or attachment part and the acetabular rim extension part (Figure 2). The attachment part was designed with a porous inner shell (1 mm sized Dode-Medium unit cell) and incorporated four screw holes and an additional ventral ilium flange for ease of positioning. A 2.7 or 3.5 mm stainless steel cortical screw (DePuy Synthes, Raynham, MA, United States) and three 2.7 or 3.5 mm stainless steel locking screws (DePuy Synthes) were used for the fixation of the implant. All screws were placed bicortically. The acetabular rim extension part was designed to extend the Norberg Angle (NA) 25–35 degrees more according to the original NA of each hip. Additionally, the acetabular rim extension part received a 1–1.5 mm offset to allow the interposition of the hip capsule between the implant and the cartilage of the femoral head.

The implants were produced from medical grade titanium alloy Ti-6Al-4V ELI grade 23 by direct metal printing using a ProX DMP320 machine (3D Systems, Leuven, Belgium). After that, the implants were postprocessed which included an in-house stress release, CNC milling of the locking threads, mirror polishing, and ultrasonic cleaning by the implant manufacturer. Additionally, final manual cleaning and autoclave sterilization were performed by our in-house sterilization facility. The general estimated lead time in implant manufacturing was 6–8 weeks.

### Surgical procedure

The surgeries were performed by a board-certificated veterinary surgeon (BM) under the standardized general anesthesia protocol. The dogs were positioned in lateral recumbency with the treated limb up. The craniodorsal approach to the hip (34) was slightly modified to identify the landmarks for implant positioning. In brief, the skin incision was oriented cranially to the greater trochanter, and the incision was made in the superficial leaf of the tensor fascia latae muscle along the cranial border of the biceps femoris muscle. The biceps femoris muscle was retracted caudally. Superficial and middle gluteal muscles were retracted dorsally. Tenotomy of the deep gluteal muscle was performed close to the insertion, and a stay suture was preplaced. The deep gluteal muscle was retracted dorsally and carefully freed from the joint capsule and its attachment to the ilium. The insertion of the rectus femoris muscle was identified as cranial to the dorsal acetabular rim which corresponded to the curvature on the implant, just caudal to the flange, and leaving the insertion free (Figure 2B(^*^)). The ventral rim of the ilium cranial to the attachment of the rectus femoris was freed for placement of the implant's flange. A periosteal elevator was used to remove all remaining soft tissues from the iliac body, preparing for the positioning of the attachment part of the implant and stimulating bone in-growth in the porous layer. The attachment part of the shelf implant was placed at its designated site, and the rim extension part was positioned over the hip joint capsule ensuring that hip laxity was reversed. The shelf implant was fixed with three stainless locking screws (3.5 or 2.7 mm, DePuy Synthes, Raynham, MA, United States) and one cortical self-tapping screw (3.5 or 2.7 mm, DePuy Synthes, Raynham, MA, United States). The non-locking bicortical screw was fixated first to maneuver the implant to its designated (designed) location and was further fixated with three locking bicortical screws. Fluoroscopy (Philips^®^ model NZS 229, Eindhoven, the Netherlands) was used from dog 10 onward (based on ongoing insight), after placement of the first screw to confirm the correct implant position before placing the last three screws. After implantation, the range of motion of the hip joint was tested for possible impingement between the implant and the major trochanter. In addition, the hip subluxation (Ortolani) test was repeated with the implant *in situ*.

Muscle closure was started by placing 1–2 locking loop sutures on the deep gluteal tendon using polydioxanone (PDS, Ethicon Inc., Somerville, NJ, United States). The tensor fascia latae muscle was opposed and sutured to the middle and superficial gluteal muscle and the biceps femoris muscle using interrupted PDS sutures. The subcutaneous tissue was closed using interrupted sutures poliglecaprone 25 (Monocryl, Ethicon), and the skin was sutured using interrupted sutures polyamide (Ethilon, Ethicon). An immediate postoperative radiograph and CT were performed to evaluate the accuracy of the implant placement. The surgical time from skin incision until skin closure of each hip was recorded. If a single-stage bilateral procedure was performed, the right hip was operated first with the dog in left lateral recumbency. After completion of ACE-X surgery on the right hip, the dog was placed in the right lateral recumbency. Following aseptic preparation of the left hindlimb, a similar procedure as executed on the right side was performed on the left side.

### Preoperative and postoperative care

Before anesthesia, dogs were categorized using the American Society of Anesthesiologists (ASA) patient scale as 1 or 2 (scale of 1–5). Epidural anesthesia was performed in all dogs by administering morphine (0.1 mg/kg) diluted with levobupivacaine (1 ml/5 kg) in the epidural space between the seventh lumbar vertebra and sacral bone. An indwelling Foley urinary catheter was placed for 12–24 h in all dogs. Cefazolin 20 mg/kg was injected intravenously 30 min before incision and every 90 min perioperatively. A non-steroidal anti-inflammatory drug (carprofen 2 mg/kg orally twice daily) and gabapentin (10 mg/kg orally every 8 h) were prescribed for continued analgesia at home for 14 days. In hyperactive dogs, trazadone hydrochloride (2–5 mg/kg, orally, 1–2 times per day) was also given for 7–14 days. Following surgery, all dogs were hospitalized for 24 h. Dogs were allowed to bear weight on the treated leg(s) immediately after surgery without abdominal support. When at home, incremental supervised activity on the leash (no running, playing, or jumping) was recommended for 6 weeks. The skin sutures were removed 14 days postoperatively. Professional physiotherapy and/or hydrotherapy were advised 2 weeks after surgery.

### Gait analysis

Before surgery, at 1.5 and 3 months after surgery, ground reaction forces (GRFs) were measured using a quartz crystal piezoelectric force plate (Kistler type 9261, Kistler Instrument AG, Winterthur, Switzerland) together with the Kistler 9865E charge amplifiers as described previously (35, 36). The force plate was 60 cm wide and 40 cm long and was mounted at the same level as the floor surface in the center of an 11-m long walkway. The force plate surface was adjusted to two smaller sizes as follows: 60^*^30 cm and 60^*^25 cm depending on the body length of the dog. GFRs were measured in mediolateral (Fx), craniocaudal (Fy), and vertical (Fz) directions. The dogs were walked over the force plate at a constant speed, and the velocity was measured by two photocells mounted 3 m apart. Measurements in which both the ipsilateral thoracic and pelvic limbs had contact with the plate were included. A minimum of 4–10 valid trials from each limb were kept for data analysis. All forces were normalized with respect to the dog's body weight. The breaking and propulsion forces from Fy direction, peak vertical force (PVF), vertical impulse (VI) and pelvic (P), and thoracic (T) index (P/T index) from Fz direction were calculated. Since most dogs in this study had bilateral ACE-X implantation and this may lead to a shift in forces from the hind limbs to the front limbs, we also calculated the P/T index of the PVF of the same side as a measure of hindlimb function.

### Owner assessment of pain-related behavior

The Helsinki chronic pain index (HCPI) questionnaire was translated into Dutch and English and supplied to the owners before surgery and at 1.5 and 3 months after surgery. As previously described (37, 38), the HCPI total score was constructed as the sum of answers to 11 questions. Each answer could be chosen from a five-point descriptive scale and was later tied to a score (0–4). Scores 0–1 fitted with normal dog behavior or locomotion while scores 2–4 fitted with abnormal behavior or locomotion. Therefore, the minimum total index score was 0, and the maximum total index score was 44. On the HCPI, the owners were asked to select only one answer to each question that best described their dog's behavior on the 11 questions including mood, play, vocalization, walking, trotting, galloping, jumping, laying down, getting up, moving after rest, and moving after major exercise. While answering the questionnaire, the values and how to compute the total score were not available to owners. The total index score for each dog was then converted to a percentage by dividing the score by the maximum index score of the answered questions for further analysis. When the dog's total index score was higher than 25%, this was interpreted as a dog being in chronic pain. The total index score lower than 13.6% was indicated as the dog is not being in pain. A total index score between 13.6 and 25% was considered inconclusive (37, 38).

### Statistical analysis

The normality of each parameter was checked by the Q-Q plot (SPSS version 28, IBM, NY, United States). The radiographic outcomes were analyzed by repeated measured ANOVA. Due to missing values, gait analysis data and HCPI data were analyzed by a generalized linear mixed model. The results of the OA score were analyzed using related samples from Friedman's two ways analysis of variance. The Kruskal–Wallis test was used to evaluate the altered osteophyte size over time between dogs with preoperative OA scores 0, 1, and 2. The Bonferroni *post-hoc* tests were performed to compare differences between time periods and OA score groups. The independent *t*-test was used to compare surgical time spent between the left and right hip. Relations of outcome results between radiography and CT, between lead time and osteophyte size, between GRFs and HCPI, and between osteophyte size and GRFs were analyzed using Pearson's and Spearman's correlations. Values with *p* < 0.05 or less were considered significant.

## Results

### Animals

Between December 2019 and March 2022, 34 client-owned dogs with 62 dysplastic hips met the inclusion criteria and participated in this study. One hip was excluded on the day of surgery due to hip laxity progression to a luxoid state. Therefore, 61 hips (34 dogs) were enrolled in this study. Breeds were identified as a mixed breed (*n* = 8), Labrador Retriever (*n* = 4), Bernese Mountain Dog (*n* = 3), Australian Shepherd Dog (*n* = 3), English Springer Spaniel (*n* = 2), Labradoodle (*n* = 2), Stabyhoun (*n* = 2), and other purebred dogs (*n* = 10). The population included 24 males and 10 females, median age of 12 months old (range 7–38 months old) and a median body weight of 27.3 kg (range 12–86 kg). A total of seven dogs were operated on unilateral hips, and 27 dogs were operated on bilateral hips. In total, seven dogs from the bilateral group received surgery in two separate sessions with a median time between sides of 92 days (56–524 days). There were 31 right hips and 30 left hips (see Supplementary Table).

### Implantation, complications, and concurrent surgical treatments

The median lead time for implant manufacturing was 74 days (range 45–174 days). The median time from implantation to the 1.5-month follow-up time marker was 45 days (range 24–65 days) and to the 3-month follow-up time marker was 94 days (68–106 days). The mean surgical time was 91 ± 22 min per hip. Mean surgical time for the right (99 ± 21 min) and left hips (84 ± 21 min) differed significantly (*p* = 0.008). A total of 33 dogs were discharged from the hospital 24 h after surgery. One dog remained hospitalized for 48 h due to ambulatory posterior paresis caused by the epidural block. Clinical signs in this dog resolved spontaneously before discharge.

Like any orthopedic surgery, ACE-X surgery carries the risk of potential complications. Potential perioperative complications are imperfect ACE-X implant/screw positioning and excessive bleeding. The potential minor postoperative complications that can be encountered are superficial surgical site infections, transient neurological deficits, and minor progression of osteoarthritic changes. The potential major complications that can be encountered are deep surgical site infections, implant failure, permanent neurological deficits, and severe increase in osteoarthritic changes leading to impairment of limb use. In this study, perioperative complications were found in three hips (4.9%) of three dogs (no 7, 9, and 14). A total of two dogs (no 7 and 9) had imperfect positioning of the implants (4–5 mm cranio-caudal deviation from planned) and received revision surgery immediately after CT imaging to correct implant placement. Based on these experiences and ongoing insight, fluoroscopy was introduced from dog 10 onward. In dog number 14, the cortical screw was judged to be marginally penetrating the joint surface, and the dog received revision surgery the next day replacing the implant and correcting the screw trajectory. After dog number 14, no further revision surgeries were performed within 24 h after ACE-X in the remainder of the dogs in this cohort. Within 3 months of follow-up, concurrent orthopedic diseases were surgically treated in two dogs. One dog received tibial plateau leveling osteotomy (TPLO) to treat cranial cruciate ligament rupture, while another dog received bilateral elbow arthrotomy to treat medial coronoid disease.

### Assessment of hip joint laxity testing (Ortolani sign)

All treated hips had a positive Ortolani sign preoperatively at intake. However, the Ortolani sign tested negative in six hips during the preoperative evaluation on the day of surgery. Postoperatively, all except two hips tested Ortolani negative; one hip had a positive Ortolani sign immediately after surgery, and another hip had a positive Ortolani sign at 1.5 months follow-up.

### Radiographic outcomes

The FCI scores of 61 treated hips by preoperative radiographs were B (*n* = 7), C (*n* = 8), D (*n* = 22), and E (*n* = 24). At 1.5 months follow-up, 33 dogs (57 hips) returned to the clinic for re-evaluation and attained ventrodorsal hip extended radiographs. The NA, LFO, and PC increased significantly directly postoperatively and at 1.5 months follow-up compared with those preoperatively at intake (Table 2).

**Table 2:** Results in dogs with hip dysplasia that underwent acetabular rim extension with a titanium shelf implant as measured preoperatively (at inclusion), postoperatively (T = 0), at 1.5 months (T = 1.5 m), and at 3 months (T = 3 m) follow-up.

**Outcome measurements**		**Preoperatively**	***T* = 0**	***T* = 1.5 m**	***T* = 3 m**	***p*-value**
Radiographic measurements (mean ± SD)	NA (°)	90 ± 12^a^	138 ± 19^b^	136 ± 19^b^	–	< 0.001^*^
	LFO (%)	29 ± 15^a^	81 ± 19^b^	82 ± 16^b^	–	0.042^*^
	PC (%)	35 ± 19^a^	85 ± 17^b^	83 ± 18^b^	–	< 0.001^*^
CT-scan measurements (mean ± SD)	NA (°)	87± 13^a^	134 ± 19^b^	-	131 ± 20^b^	< 0.001^*^
	LFO (%)	22 ± 15^a^	81 ± 16^b^	-	76 ± 19^c^	< 0.001^*^
	PC (%)	33 ± 17^a^	79 ± 21^b^	-	77 ± 20^b^	0.002^*^
Ground reaction forces (mean ± SD)	PVF (%BW)	40.86 ± 6^a^	–	38.9 ± 5.5^b^	39.21 ± 4.4^b^	0.024^**^
	P/T index	0.61 ± 0.1^a^	–	0.56 ± 0.1^b^	0.58 ± 0.1^b^	< 0.001^**^
	VI (%BW.s)	14.36 ± 2.9	–	14.04 ± 2.9	13.68 ± 3.3	0.118
	Breaking force (%BW)	6.39 ± 1.6	–	6.21 ± 1.9	6.32 ± 1.7	0.781
	Propulsion force (%BW)	6.44 ± 1.4^a^	–	6.0 ± 1.6^a, b^	5.31 ± 1.6^b^	< 0.001^**^
HCPI (%) (Mean ± SD)		31.44 ± 11.9^a^	–	20.39± 10.9^b^	17.69± 10.8^b^	< 0.001^**^

NA, Norberg angle; LFO, linear percentage of femoral head overlap; PC, percentage of femoral head coverage; SD, standard deviation; PVF, peak vertical force; P/T index, pelvic/thoracic index; VI, vertical impulse; BW, body weight; s, second.

HCPI (%) = 100% × total index score/maximum possible index score of the answered questions.

^a − c^*p*-value of < 0.05 from the Bonferroni correction, *p*-value^*^ from repeated measure analysis, *p*-value^**^ from generalized linear mixed model.

At 3 months follow-up, 23 dogs (38 hips) returned to the clinic for re-evaluation and attained CT scans of the hip joints. Like 1.5 months follow-up, the NA, LFO, and PC measurements from CT increased significantly directly postoperatively and at 3 months follow-up compared with preoperatively at intake. When LFO was compared directly postoperatively and at 3 months follow-up, a small significant decrease in LFO was noted (Table 2).

**Figure 3:**
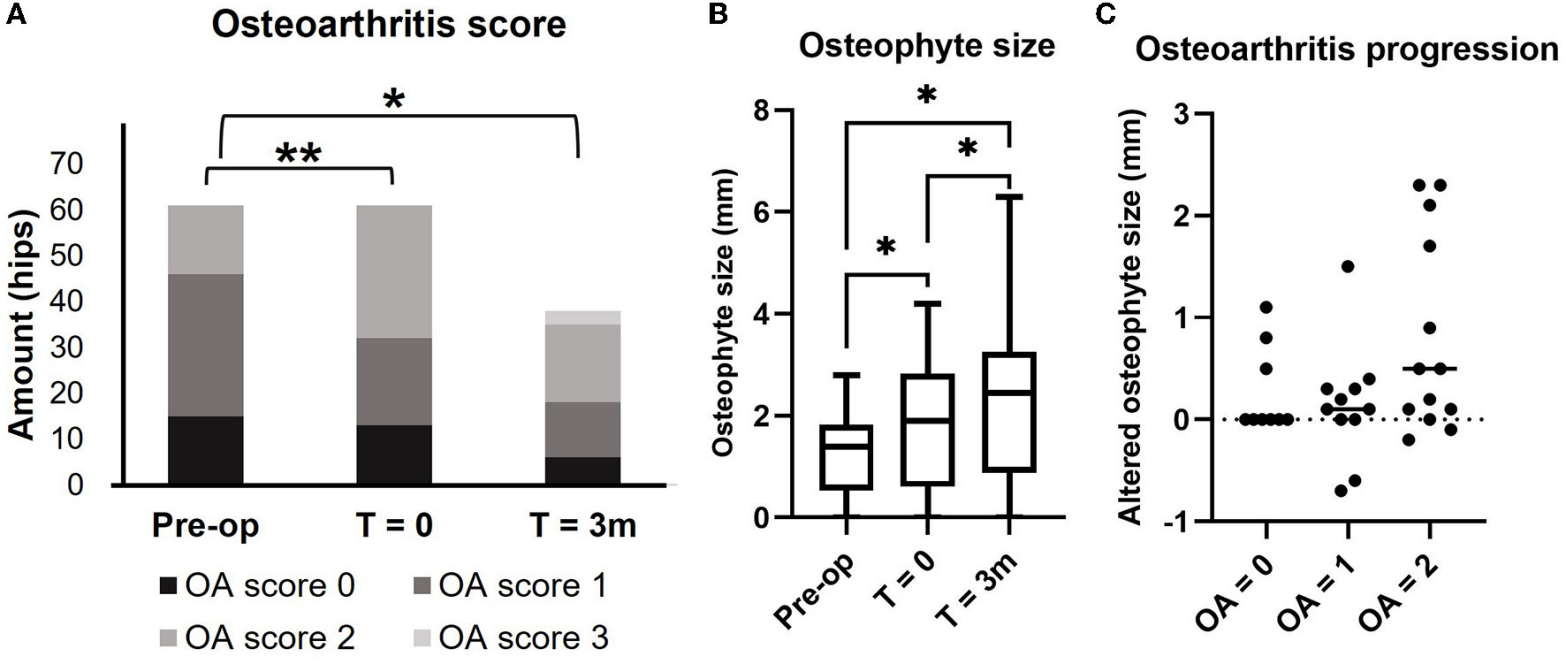
Preoperative and postoperative osteoarthritis score **(A)** osteophyte size **(B)**, and comparison of osteophyte size growth between T = 0 (postoperatively) and T = 3 months (3 m) grouped by OA score at the day of surgery (T = 0) **(C)**. Osteoarthritis score and osteophyte size were measured, respectively, on CT images of the hip joints, preoperatively at intake, T= 0 (postoperatively), and T= 3 months (3 m). **(A)** Each bar represents the number of hips in each OA score at each time point. **(B)** Each bar represents the mean osteophyte size ± SD. **(C)** Each dot represents the changes of osteophyte size between T = 0 and T = 3 m. *p-value of < 0.05 using the Bonferroni correction for multiple tests, **p-value of < 0.05 using related samples for Friedman's test.

The OA scores from the preoperative CT planning at intake (61 hips) were 0 (*n* = 15), 1 (*n* = 31), and 2 (*n* = 15), and they significantly changed to OA scores 0 (*n* = 13), 1 (*n* = 19), and 2 (*n* = 29) at the day of surgery (*T* = 0) (*p* < 0.001). Median OA score of 3-month follow-up (38 hips) (2; range 0–3) differed significantly from preoperative OA score at intake (1; range 0–2) (*p* = 0.002) but did not significantly differ from the day of surgery (T = 0) (1; range 0–2) (*p* = 0.325) (Figure 3A). Mean (±SD) osteophyte size increased significantly over time from preoperative CT at intake (1.26 ± 0.84 mm) to the day of surgery (T = 0) (1.86 ± 1.28 mm) and at 3 months follow-up (2.35 ± 1.73 mm) (*p* < 0.05) (Figure 3B). The median increase in osteophyte size from postoperative CT at the day of surgery (T = 0) to 3 months follow-up CT (T = 3 m) (38 hips) was not significantly different between the three groups of OA scores 0 (0 mm, range 0–1.1 mm), 1 (0.1 mm, range −0.7 to 1.5 mm), and 2 (0.5 mm, range −0.2 to 2.3 mm) (*p* = 0.074) (Figure 3C).

**Figure 4:**
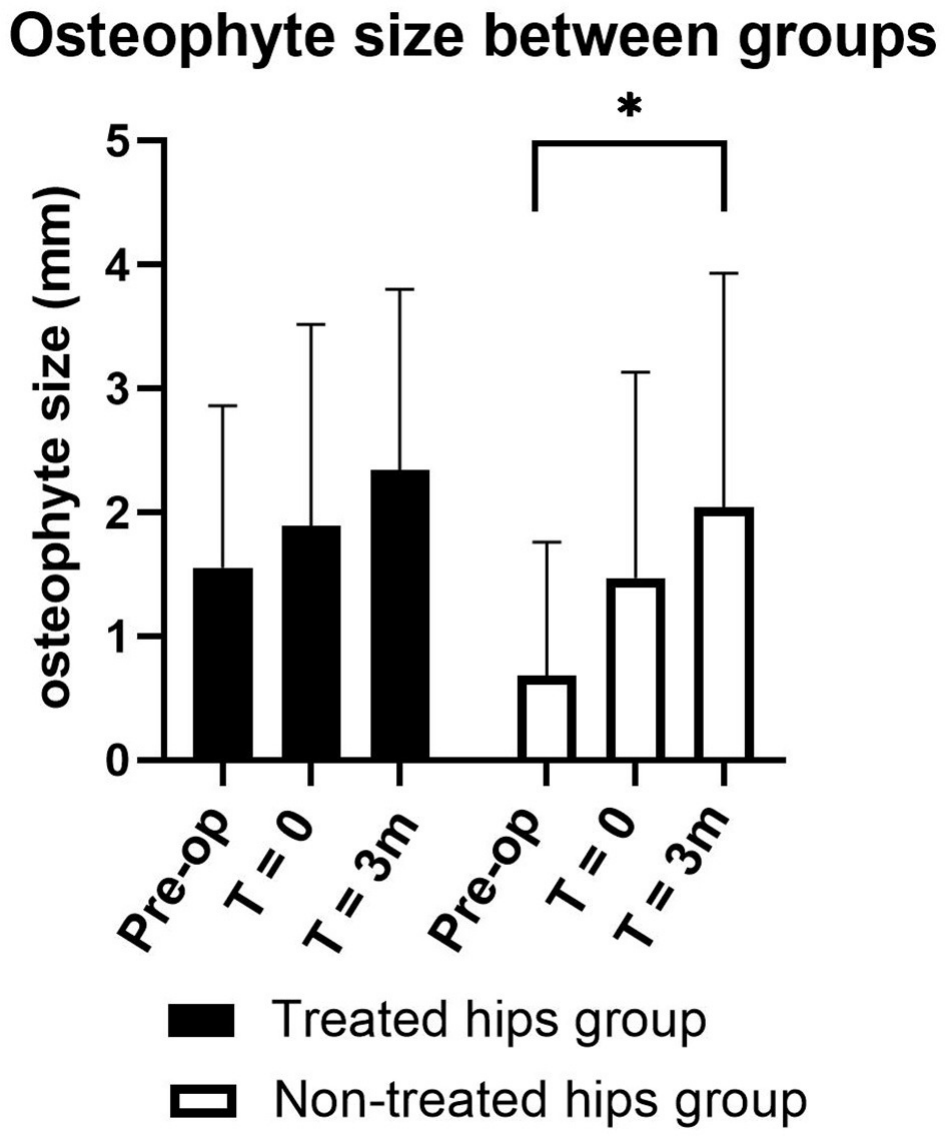
Preoperative and postoperative osteophyte size compared between treated hip (black bar) and non-treated hip (white bar) group for 3 months (m) postoperative follow-up in 12 dogs with bilateral hip dysplasia that underwent unilateral acetabular rim extension surgery. Each bar represents the mean osteophyte size ± SD. *p-value < 0.05 using the Bonferroni correction for multiple tests.

At 3 months follow-up, 12 dogs with bilateral HD that underwent unilateral ACE-X surgery returned to the clinic and attained CT scans. There was no significant difference in osteophyte size between the treated and the non-treated hip group. In the non-treated hip group, osteophyte size increased significantly 3 months postoperatively (2.04 ± 1.89 mm) compared with preoperatively (0.68 ± 1.08 mm) (*p* = 0.004), whereas this change was not significant in the treated hip group. The change in osteophyte size between the day of surgery (T = 0) and at 3 months follow-up (T = 3) was not significant for both groups (Figure 4).

### Kinetic gait analysis

Ground reaction forces were measured preoperatively (*n* = 48) at 1.5 months (*n* = 47) and 3 months (*n* = 31) follow-up. Significant differences were noted in the peak vertical force (PVF) and P/T index between preoperative evaluation and at 1.5 and 3 months follow-up. In addition, the propulsion force differed significantly between the preoperative evaluation and 3 months of follow-up (Table 2).

### Owner assessment of pain-related behavior

Helsinki chronic pain index (HCPI) questionnaires were collected from the owners pre-operatively (*n* = 25) at 1.5 months (*n* = 20) and 3 months follow-up (*n* = 14). The questionnaires were evaluated for each individual dog regardless of whether they had unilateral or bilateral ACE-X implantation. Significant differences were found in HCPI total percentage when comparing preoperative evaluation (31%) with 1.5 months (20%) and 3 months (17%) follow-up (Table 2).

### Correlations

A statistically significant positive correlation was found between measurements of ventrodorsal hip extended radiographs and coronal CT images of the hip joints on NA (*r* = 0.95), LFO (*r* = 0.89), and PC (*r* = 0.91). A statistically significant negative correlation was found between an increase in osteophyte size and VI (*r* = −0.33) and propulsion force (*r* = −0.39).

## Discussion

Canine developmental hip dysplasia has been shown to alter hip mechanics and promote the development of osteoarthritis, which can lead to impairment of hip joint mobility and chronic pain. This study intended to answer the following questions: (1) Does acetabular rim extension with a personalized titanium shelf implant improve femoral head coverage and normalize hip laxity and reduce osteoarthritis progression in dogs clinically affected by hip dysplasia in the short term? (2) Are the short-term functional results favorable, particularly in relation to chronic pain and limb use?

The postoperative morbidity after ACE-X surgery was low, the complication rate after 3 months follow-up was 4.9%, and most of these complications were minor consisting of imperfect implant positioning and misdirection of a screw, which was solved during the ongoing study by perioperative imaging. In TPO, the complication rates varied between 7 and 70% (14–17) and consisted of minor and major complications such as loosening of the screws, surgical wound infections, pelvic canal narrowing, excessive head coverage by the acetabular roof and subsequent impingement of the femoral head, delayed healing of the iliac and ischial osteotomies, peripheral nerve injury, loss of alignment of the axis of the ilium, and high morbidity, especially after simultaneous bilateral surgery. In DPO, the complication rates vary between 8.3 and 20.7% (8, 10) and mainly consist of implant failure or loosening and incomplete fracture of the ischial table. In ACE-X surgery for 3 months follow-up, no implant failures, no wound infection, and no neurological deficits except for one case with 24-h paraparesis due to epidural analgesia block were seen. Most likely, the dural sac was punctured and/or the analgesic agent migrated cranially to the (thoraco) lumbar region causing spinal anesthesia which is a reported complication during lumbosacral epidural anesthesia (39).

In this study, the mean surgical time for unilateral ACE-X implantation (91 ± 22 min) was shorter than that for TPO (107 ± 39 min) (40, 41). This is promising since the surgical time for ACE-X also included the learning curve time for the first hips and intraoperative imaging, and it is expected that ACE-X surgical time will further decrease with continued experience. The duration of unilateral ACE-X implantation on the right side (99 ± 21 min) was prolonged compared with the left side (84 ± 21 min). There are several explanations that may have contributed to this difference. One explanation is the effect during the tightening of the first screw on the acetabular rim extension part of the implant. Since all screws turn clockwise when they are tightened, the rim extension of the ACE-X implant may turn slightly clockwise on the right side (thereby minimally losing its perfect positioning), whereas on the left side, clockwise tightening of the first screw will turn the ACE-X implant to its perfect position. For this reason, fluoroscopy time on the right side may logically have taken more time than on the left side before perfect positioning was reached on the right side. Since surgery was performed in an academic institution with different surgical assistants (students, interns, and residents) in each dog, every bilateral procedure also included an educational part during the first (right) side, whereas on the second (left) side, the workflow was known to the surgical team contributing to a shorter surgery time. A major factor that contributed to shorter surgery times during the cohort study was improved exposure to the surgical field, especially the dorsal acetabular rim. Following tenotomy of the deep gluteal muscle and elevating the muscle from the hip capsule and its ilial insertion, the exposure of the dorsal acetabular rim was only sufficiently achieved by femoral abduction and flexion–exorotation of the stifle thereby releasing the tension on the complete gluteal muscle complex and increasing the surgical view on the dorsal acetabular rim and facilitating positioning of the implant during screw insertion.

Our case series consisted of a very different age-related study population since dogs in the age of 7–38 months old were enrolled. However, no adaptations in surgical technique, surgical approach, or surgical equipment were necessary when performing this surgery in skeletally immature or mature dogs. In addition, we did not experience any significant difference in terms of the difficulty of surgical approach or implant fixation between skeletally immature and mature dogs.

In this study, we assessed the increase of femoral head coverage by the implant by measuring NA, LFO, and PC. Based on the improvement of the mean CT NA (131°), LFO (76%), and PC (77%) at 3 months follow-up exceeding the minimum standard value of NA (>105°), LFO (>50%), and PC (>50%) (33, 42), the present study demonstrated that ACE-X implantation improved and provided for sufficient femoral head coverage. Furthermore, despite the small decrease of LFO at 3 months of follow-up (76%) compared with postoperatively (81%), the value at 3 months of follow-up was still higher than the standard minimal value (>50%). In addition, a minimal decrease of NA and PC (although not significant) was found at 3 months follow-up compared with postoperatively which was similar to the study of Vezzoni et al. (8). In the latter study, a minimal non-significant reduction of NA and PC at 2 months after DPO was found compared with 1 month and direct postoperatively. However, Petazzoni et al. (12) reported a minimal non-significant increase in NA, PC, and LFO at 8 weeks after DPO compared with direct postoperatively. A strong correlation was found between radiographic measurements and CT measurements indicating that one of the imaging techniques can be used for determining femoral head coverage after ACE-X implantation.

Like DPO/TPO (11, 14, 18), ACE-X implantation is intended to prevent or delay the progression of osteoarthritis. The inclusion of dogs in this cohort study was performed on the preoperative radiograph by the referring veterinarian (no or minimal OA), whereas OA scoring was conducted on the preoperative planning CT. From preoperative CT, the majority of the hips included in our study had minimal to no OA; however, < 25% had moderate OA. The time period between inclusion, planning CT, and time of surgery (i.e., lead time) was relatively long in some dogs. It was very clear that the OA score and osteophyte size already increased significantly during the lead time. Since the median design and manufacturing lead time before ACE-X implantation were more than 2 months, the increase in OA score and osteophyte size directly postoperatively (T = 0) compared with preoperative planning may be explained by continued hip laxity. Therefore, it is advised for future studies to improve the workflow efficiency between clinical diagnosis and delivery of the 3D print shelf implant in order to minimize the lead time as much as possible. Within this context, the progression of osteophyte size over 3 months follow-up in the dogs with OA score 2 (0.5 mm) was further advanced than in those with OA score of 0 or 1 indicating that ACE-X implantation is more suitable for dogs without osteoarthritis. This is in line with observations in DPO/TPO treatment where dogs with more advanced osteoarthritis preoperatively are more likely to have an unfavorable clinical outcome after surgery (43). Noteworthy, this is the first study that examined the progression of OA using CT, and it is anticipated that CT examination of the hips is far more detailed and accurate than radiographic examination used in other studies documenting the outcome of other hip surgical techniques like DPO/TPO or JPS. The previous studies showed that CT improved efficiency in the diagnosis of hip and elbow dysplasia and improved early identification of dogs predisposed to hip and elbow joint osteoarthritis (44, 45).

Despite an increase in the OA score and osteophyte size at different follow-up periods in the cohort of dogs, HCPI scores decreased, indicating a reduction in chronic pain after ACE-X implantation. The finding that clinical lameness and radiographic changes are not correlated was also shown previously by Innes et al. (46). The preoperative total HCPI score was 31%, indicating that most dogs were in chronic pain, and this score decreased to 18% at 3 months follow-up. Despite that the HCPI score was in the inconclusive zone at 3-month follow-up, this significant reduction indicated the ACE-X implant improved the dogs' wellbeing in the short term.

In contrast to the decrease in HCPI score at follow-up indicating clinical improvement, small significant changes were found in force plate data after ACE-X surgery still indicating somewhat impaired gait. A reduction in PVF, P/T index, and propulsion force was found at a 1.5-month follow-up. In addition, there was a reduction in propulsion force at 3 months follow-up when compared with the baseline and 1.5 months follow-up, indicating less weight bearing of the treated limbs. In this study, the force plate results may be explained by the low baseline values of PVF (40.86 ± 5.97 %BW) and P/T index (0.61 ± 0.1) which almost resemble the values of healthy dogs (PVF 40.4%, P/T index 0.63) (35), making it difficult to detect large changes in gait improvement. In addition to that, the follow-up period may be too short to detect significant improvements in limb function; muscle and tendon healing can require several months to a year after surgery, and insufficiently healed tissues might contribute to a decrease in limb use (47). Like our study, McLaughlin et al. (48) found a reduction of vertical forces at week 5 after TPO, but values gradually increased until the last visit at week 28. A negative correlation was found between osteophyte size and propulsion force and between osteophyte size and vertical impulse indicating that OA progression may influence limb function. The discrepancy between the reduction in HCPI score and lack of significant improvement of force plate data at short-term follow-up might have been caused by the relatively low walking speed used to evaluate the dogs on the force plate since most clinical signs of dogs with hip dysplasia are related to exercise (49). In addition, the force plate gait analysis only investigates a limited part of the complete motion spectrum of dogs that includes walking, trot, galop, jumping, playing, running, getting up, and lying down. In addition, owners may not be able to detect changes in subtle lameness or they may have been biased in their responses shifting their internal values since they were aware that their dogs have undergone (expensive) treatments which is inherent to the unblinded nature of our study (50–52). Moreover, Essner et al. (51) found some limitations of the HCPI questionnaire in two out of 11 questions; one mood item targets play behavior, which may be associated with age, breed, and personality of the dog. Another item is vocalization, which was less correlated with chronic pain. Future studies should consider including force plate gait analysis with e.g., trotting gait after exercise, lameness scoring by blinded veterinarians, and other validated questionnaires, e.g., the canine brief pain inventory score (CBPI) or the Liverpool Osteoarthritis in Dogs (LOAD) questionnaire.

The Ortolani test is the most common palpation technique that is used in veterinary medicine to diagnose functional hip joint laxity in young dogs (4–12 months of age) and showed sufficient sensitivity in the prediction of the development of canine hip dysplasia at a later age (53, 54). Radiographic views of the dorsal acetabular rim (DAR views) and distraction-based radiographic techniques can aid in the determination of hip joint laxity. However, due to the shape of the ACE-X implant, postoperative DAR projections were not possible, and the PennHIP method was unavailable at our institution. Therefore, in this study, we monitored hip joint laxity using Ortolani's testing. In our case series, all hips except two became Ortolani test negative after ACE-X implantation indicating that overall, this procedure effectively reduced hip laxity in the short term. One dog with a postoperative positive Ortolani hip joint had insufficient coverage by the implant; the measured postoperative NA was lower than the minimum standard value of 105 degrees. The other dog had concurrent lateral patellar luxation grade 2 of the treated limb which might have contributed to atrophy of the quadriceps muscle group, one of the stabilizers of the hip joint (55, 56). In contrast to DPO, the positive Ortolani sign of most dogs in ACE-X surgery immediately became negative postoperatively, while, in DPO, it only slowly disappeared within 6 months postoperatively (8) and thus may still have contributed to persistent chronic pain after pelvic osteotomy. On the day of ACE-X surgery, six hips tested Ortolani negative while they tested positive at the preoperative evaluation for CT planning which was suspected to be the result of the long lead time (74 days; range 46–158 days) and the progression of OA, but no correlation between lead time and osteophyte size was found in the case series. Other secondary changes around the joint such as periarticular fibrosis might have reduced palpable laxity and might explain these differences between the baseline day of CT planning and the day of surgery. Nevertheless, in dogs with hip laxity due to HD, the rapid osteophyte formation due to cartilage damage and reactive soft tissue changes around the joint necessitate a short lead time between planning CT and the surgery date in order not to lose momentum and efficacy of ACE-X treatment.

Compared with DPO/TPO, the craniodorsal approach of the hip joint with the extension of the acetabular rim using the ACE-X implant was less invasive and less complex than performing two or three pelvic osteotomies in DPO/TPO. Moreover, the ACE-X surgery created immediate hip joint stability; therefore, it is anticipated that early postoperative rehabilitation may be achieved. After initial positive clinical experiences with unilateral ACE-X surgeries with fast gait recovery and low postoperative morbidity, we decided for the benefit of the dog to perform bilateral ACE-X procedures in one operative session. Within this context, bilateral DPO/TPO in a single session is more challenging, has a high complication risk, and is contraindicated in giant dogs (8, 14, 43). It is tempting to hypothesize that because ACE-X has a one-surgical approach strategy and there is no need for triple/double incisions and bone cuts as in TPO/DPO (8, 10), it may facilitate a faster learning curve for specialized veterinary surgeons.

In conclusion, an acetabular rim extension with a personalized 3D-printed titanium shelf implant improves femoral head coverage and normalizes hip joint laxity. Shelf arthroplasty with ACE-X implants has no need for pelvic osteotomies and is associated with low postoperative morbidity and fast gait recovery allowing bilateral procedures in one operative session. The less demanding surgical technique, preservation of pelvic geometry, and almost no short-term postoperative complications make ACE-X surgery an attractive alternative to DPO/TPO. Further studies are required to evaluate the long-term efficacy of ACE-X surgery in dogs with hip dysplasia, with special emphasis on the preservation of hip congruency, elimination of hip joint laxity, and assessment of osteoarthritis progression. For this purpose, long-term follow-up examinations using the PennHIP method or laxity index using the Vezzoni-modified Badertscher distension device are required to objectively determine the outcome of ACE-X surgery. Moreover, improvement of limb use, radiographic osteoarthritis progression, and the accuracy of implant placement and implant migration over time should be evaluated.

## Data availability statement

The original contributions presented in the study are included in the article/Supplementary material, further inquiries can be directed to the corresponding author.

## Ethics statement

Ethical review and approval was not required for the animal study because all dogs in this study received the standard of care, therapeutic, and diagnostic work-up. Written informed consent was obtained from the owners for the participation of their animals in this study.

## Author contributions

BM, MT, BW, HW, TH, and RS contributed to the conception and design of the study. BM performed surgeries. IK and BM collected data. KW and JM designed the implants. IK organized the database, performed the statistical analysis, and wrote the first draft of the manuscript. BM, JM, KW, and FV wrote sections of the manuscript. All authors contributed to the manuscript revision and read and approved the submitted version.
