# Supplementary material for: Acetabular rim extension using a personalized titanium implant for treatment of hip dysplasia in dogs: short-term results
Source: Front Vet Sci. 2023 Apr 20;10:1160177. doi: 10.3389/fvets.2023.1160177 (PMC10157081; doi:10.3389/fvets.2023.1160177)
Supplement: Supplementary file 1 [file Table_1.DOCX]

**Acetabular rim extension using a personalized titanium implant for treatment of hip dysplasia in young dogs: short term results**

Irin Kwananocha, Koen Willemsen, Joëll Magré, Harrie Weinans, Ralph J.B. Sakkers_,_ Thijs How, Femke Verseijden, Marianna A. Tryfonidou, Bart C.H. van der Wal and Björn P. Meij^*^

^*^**Correspondence:** Björn P. Meij: b.p.meij@uu.nl

**Supplementary Table: Demographics of dogs included in this study.**

| Case No. | Breed | Sex | Side of surgery | Age at surgery (month) | Body weight (kg) |
| --- | --- | --- | --- | --- | --- |
| 1 | Labrador Retriever | F | bilateral^a^ | 12/14^b^ | 27.3/27.3^c^ |
| 2 | Spanish Mastiff | F | bilateral^a^ | 24/32^b^ | 65/71.5^c^ |
| 3 | Labradoodle | M | right | 12 | 21 |
| 4 | Stabyhoun | M | left | 17 | 22 |
| 5 | Shiba Inu | M | bilateral^a^ | 22/38^b^ | 17.5/18.9^c^ |
| 6 | Mixed breed | M | bilateral^a^ | 10/22^b^ | 35.8/40^c^ |
| 7 | Saint Bernard | M | bilateral^a^ | 12/15^b^ | 86/86^c^ |
| 8 | Bernese Mountain dog | M | right | 19 | 36 |
| 9 | Labrador Retriever | F | bilateral | 13 | 29.8 |
| 10 | Bouvier des Flandres | M | bilateral | 9 | 45 |
| 11 | Mixed breed | M | bilateral^a^ | 20/23^b^ | 14.5/15.4^c^ |
| 12 | German Shepherd dog | M | bilateral | 8 | 26.8 |
| 13 | Mixed breed | F | bilateral | 12 | 24.7 |
| 14 | Australian Shepherd dog | F | bilateral | 11 | 19 |
| 15 | Australian Shepherd dog | F | bilateral | 11 | 18.8 |
| 16 | New Foundland | M | bilateral^a^ | 9/12^b^ | 38.6/43.7^c^ |
| 17 | Mixed breed | M | bilateral | 11 | 21.3 |
| 18 | Mixed breed | F | bilateral | 17 | 17.3 |
| 19 | Pyrenean Sheepdog | M | bilateral | 15 | 12.2 |
| 20 | Labrador Retriever | M | bilateral | 10 | 31.2 |
| 21 | Mixed breed | M | left | 15 | 32.2 |
| 22 | Border Collie | F | right | 7 | 13.8 |
| 23 | Stabyhoun | M | bilateral | 11 | 27.9 |
| 24 | English Springer Spaniel | F | left | 11 | 15.1 |
| 25 | Appenzeller Sennenhund | M | right | 14 | 24 |
| 26 | Collie | M | bilateral | 10 | 29.8 |
| 27 | Labrador Retriever | M | bilateral | 10 | 25.1 |
| 28 | English Springer Spaniel | M | bilateral | 12 | 19.1 |
| 29 | Leonberger | F | bilateral | 8 | 41.9 |
| 30 | Bernese Mountain dog | M | bilateral | 12 | 35.6 |
| 31 | Australian Shepherd dog | M | bilateral | 12 | 28 |
| 32 | Bernese Mountain dog | M | bilateral | 10 | 50 |
| 33 | Mixed breed | M | bilateral | 16 | 24.4 |
| 34 | Labradoodle | M | bilateral | 21 | 20.4 |

M: male, F: female

^a^ bilateral hip surgery in separate sessions

^b^ age at the first and at the second surgery

^c^ body weight at the first and at the second surgery
